# The incidence of antibiotic resistance within and beyond the agricultural ecosystem: A concern for public health

**DOI:** 10.1002/mbo3.1035

**Published:** 2020-07-25

**Authors:** Chidozie D. Iwu, Lise Korsten, Anthony I. Okoh

**Affiliations:** ^1^ SAMRC Microbial Water Quality Monitoring Centre University of Fort Hare Alice South Africa; ^2^ Applied and Environmental Microbiology Research Group Department of Biochemistry and Microbiology University of Fort Hare Alice South Africa; ^3^ Department of Plant and Soil Sciences Faculty of Natural and Agricultural Sciences University of Pretoria Pretoria South Africa

**Keywords:** agro‐ecosystem, antibiotic resistance, environment, food safety, public health

## Abstract

The agricultural ecosystem creates a platform for the development and dissemination of antimicrobial resistance, which is promoted by the indiscriminate use of antibiotics in the veterinary, agricultural, and medical sectors. This results in the selective pressure for the intrinsic and extrinsic development of the antimicrobial resistance phenomenon, especially within the aquaculture‐animal‐manure‐soil‐water‐plant nexus. The existence of antimicrobial resistance in the environment has been well documented in the literature. However, the possible transmission routes of antimicrobial agents, their resistance genes, and naturally selected antibiotic‐resistant bacteria within and between the various niches of the agricultural environment and humans remain poorly understood. This study, therefore, outlines an overview of the discovery and development of commonly used antibiotics; the timeline of resistance development; transmission routes of antimicrobial resistance in the agro‐ecosystem; detection methods of environmental antimicrobial resistance determinants; factors involved in the evolution and transmission of antibiotic resistance in the environment and the agro‐ecosystem; and possible ways to curtail the menace of antimicrobial resistance.

## Graphical abstract

This study outlines an overview of the discovery and development of commonly used antibiotics; the timeline of resistance development; drivers and hotspots of environmental antimicrobial resistance; transmission routes of antimicrobial resistance in the agro‐ecosystem; detection methods of environmental antimicrobial resistance determinants; factors involved in the evolution and transmission of antibiotic resistance in the environment and the agro‐ecosystem; and possible ways to curtail the menace of antimicrobial resistance.

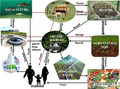

## 1 INTRODUCTION

The pervasive distribution of antibiotic resistance determinants in bacteria is responsible for the failure in the effectiveness of antibiotics used for treating infections caused by an animal or human pathogens (Brown & Wright, 2016). For the past two decades, the discovery of new antibiotics to counter the development of antimicrobial resistance (AR) has rapidly declined (Wendlandt et al., 2015). Also, a complex relationship exists between the misuse of antibiotics and the evolution and distribution of antibiotic resistance in the human, plant, animal, and environmental microbiota (Wendlandt et al., 2015). Therefore, the evolution of antibiotic resistance can be correlated with selective pressures caused by the antibiotics that are indiscriminately used (Zhang, Gu, et al., 2018).

The effects associated with the misuse of antibiotics in veterinary and the medical sectors alongside the release of antibiotic‐resistant bacteria (ARB) and antibiotic resistance genes (ARGs) from sources where the anthropogenic activity is high are currently considered as an environmental challenge (Berendonk et al., 2015). Unlike the majority of chemicals and organic contaminants in the environment that can easily degrade or diminish in concentration over time, microbial contaminants and ARGs can persist and disperse in the environment by either multiplying within their hosts, be dispersed to other bacteria, or be subjected to other evolutionary processes, thus posing serious human, animal, and environmental health risks (Berendonk et al., 2015). However, due to the ability of some antibiotics to be degraded, some studies have revealed a weak correlation between the distributions of ARB, ARGs, and corresponding antibiotics in the environment including wastewater, manure, and the solid waste materials (Gao, Munir, & Xagoraraki, 2012; Ji et al., 2012; Pei, Kim, Carlson, & Pruden, 2006; Wu, Huang, Yang, Graham, & Xie, 2015).

Antimicrobial resistance can spread in the environment through horizontal and vertical gene transfers via mutation and recombination, and most ARGs transferred to pathogenic bacteria through horizontal gene transfer originate from bacteria living in the environment. Thus, the importance of routine investigations of environmental bacteria is important in providing predictive information on the development of AR in the environment (Zhang, Gu, et al., 2018). The circumstance in the agricultural ecosystem is more complex, as various agricultural activities and niches may uniquely influence the tenacity and spread of antibiotic resistance (Mafiz et al., 2018). In the agricultural ecosystem, antibiotics, ARB, and ARGs are spread within the complex system and eventually to the food web. The use of antibiotics in health clinics, farm animals, and aquaculture has encouraged the extension of AR reservoirs to other sections of the agro‐ecosystem and eventually back to humans through the release of wastewater effluents to receiving watersheds, spreading of bio‐solids and manure to soil, and irrigation of farm produce using graywater (Binh, Heuer, Kaupenjohann, & Smalla, 2008; Davies &Davies, 2010; Knapp, Dolfing, Ehlert, & Graham, 2010; Munir & Xagoraraki, 2011). This causes unpredicted consequences to the health of humans (Martinez, 2009; Zhang, Marrs, Simon, & Xi, 2009) and suggests that antibiotic resistance is both a public and environmental health challenge (Hu, Gao, & Zhu, 2017).

The World Health Organization (WHO) reported that ARB on the farms can contaminate vegetables and fruits as DNA fingerprinting has confirmed a link between ARB isolated from sick people and an agricultural source (WHO, 2017). Despite the importance of the agricultural ecosystem in the provision of food, scant information is available on the dissemination routes of antibiotic resistance within the complex nexus of the agro‐ecosystem and eventually to humans through plant and animal foods. This review, therefore, attempts to describe the possible transmission routes of antibiotics, ARB, and ARGs through the various sections of the agro‐ecosystem including aquaculture, farm animals, aquatic environments, manure, agricultural soil, irrigation water, plant produce, and back to humans, together with their associated health and economic implications.

## 2 HISTORICAL PERSPECTIVES

### 2.1 Discovery and development of antibiotics

Antimicrobials are compounds of either chemical, natural (from fungi or bacteria), or semi‐synthetic origin, which terminate or inhibit microbial growth with minimal or no damage to the host (Linares‐Otoya et al., 2017). The era of antibiotics started in 1928 when Alexander Fleming accidentally discovered penicillin by noticing that *Penicillium notatum*extruded a substance that inhibited the growth of *Staphylococcus*. Florey and Chain further purified it for use, and this led to the development of different classes of penicillin (Penesyan, Gillings, & Paulsen, 2015). Antibiotics were then described by the Nobel Laureate Selman Waksman, a co‐discoverer of streptomycin as “natural compounds of microbial origin” after realizing that fungi and bacteria isolated from the environment could produce metabolites that can remarkably treat the human infection with minimal side effects (Brown & Wright, 2016). His strategy later termed the “Waksman platform,” involved screening of the spore‐forming Actinomycetes and other soil‐dwelling bacteria, for metabolite secretion that could subdue microbial growth (Lewis, 2012). Between the 1950s and 1970s, the Waksman concept introduced the “golden era of antibiotic discovery,” a time when newer classes of nonsynthetic antimicrobials were discovered (Brown & Wright, 2016). However, by mid‐1960s, it became more challenging to find newer and more effective antibiotics using this concept, mainly because these antibiotics were the products of microbial growth that required specific environmental conditions. This resulted in the emergence of resistance to early antibiotics because of the toxicological and pharmacological drawbacks associated with them (Brown & Wright, 2016). This prompted the introduction of the medicinal chemistry era in 1975 that involved the innovative production of synthetic versions of antibiotics. These antibiotics had exceptional efficacy by increasing the spectrum of activity of antibiotics at low doses, preventing the occurrence of resistance and improving the general application of antibiotics (Brown & Wright, 2016). Over time, these agents began to exhibit downstream pleiotropic and complex effects on bacterial cells, forcing microbes to evolve survival strategies (Brown & Wright, 2016). Therefore, the innovative production of synthetic antibiotics failed to produce agents that are more effective than antibiotics of natural origin in the long term. In the near future, unconventional strategies that use natural antibiotics including their semi‐synthetic versions to create more narrow‐spectrum antibiotics and associated diagnostics may need to be implemented (Brown & Wright, 2016).

### 2.2 Antibiotics: Benefits, classes, and the timeline for the development of antibiotic resistance

Antibiotics play crucial roles in not only saving the lives of patients but also attaining great achievements in human and animal medicine and surgery (Gould, Bal, Gould, & Bal, 2013). Antibiotics help to prevent and treat infections in patients that have long‐term ailments such as tuberculosis, diabetes, kidney disease, and rheumatoid arthritis as well as patients that have undergone major surgeries such as joint replacements, cardiac surgery, or organ transplants or those receiving chemotherapy treatments (Ventola, 2015). Antimicrobials help in prolonging the life expectancy of people by improving the recovery from infections caused by bacteria (Piddock, 2012; Rossolini, Arena, Pecile, & Pollini, 2014). They also decrease the rate of morbidity and death resulting from food‐borne and poverty‐associated infections, especially in underdeveloped and developing countries with poor sanitation (Rossolini et al., 2014). Antibiotics vary significantly in their range of activity. Some are “narrow‐spectrum,” implying that they are only toxic to a limited number of pathogens. Others are “broad‐spectrum,” attacking several pathogens. Antibiotics act against bacteria through the following pathways: (a) interruption of cell wall synthesis and repair, (b) modification of cell membranes, (c) interference with protein synthesis (50S rRNA and 30S rRNA), (d) prevention of the synthesis of nucleic acid, and (e) antagonistic and antimetabolites characteristics (Grenni, Ancona, & Caracciolo, 2018). They generally exhibit either static or cidal effect against their target organisms. Many antimicrobial medications are available to combat infections and increase animal productivity. Nonetheless, they fall under a few classes according to their modes of action, routes of administration, toxicity, and adverse effects, as summarized in Table 1.

**TABLE 1:** Timeline of the discovery, introduction, and development of resistance to commonly used antibiotics (Adapted from Jayaraman, 2009; Lewis, 2013)

Antibiotics class	Example (s)	YOD	YOI	YOR	The mechanism (s) of action	Resistance mechanism(s)
β‐lactams	Cephalosporins, Penicillins, Cefotaxime, Monobactams, Carbapenems	1928	1938	1945	Cell wall biosynthesis inhibition	Cleavage by β‐lactamases, ESBLs, Carbapenemases, Cefotaximases, and altered Penicillin‐binding proteins
Aminoglycosides	Gentamicin, streptomycin	1943	1946	1946	Protein synthesis inhibition	Ribosomal mutations, enzymatic modification, 16S rRNA methylation, and efflux pumps
Phenicols	Chloramphenicol	1946	1948	1950	Inhibition of protein synthesis	Mutation of the 50S ribosomal subunit, reduced membrane permeability, and elaboration of chloramphenicol acetyltransferase
Macrolides	Erythromycin, azithromycin	1948	1951	1955	Alteration of protein synthesis	Ribosomal methylation
Tetracyclines	Minocycline, tigecycline	1944	1952	1950	Alteration of translation	Mainly efflux
Rifamycins	Rifampin	1957	1958	1962	Alteration of transcription	Altered β‐subunit of RNA polymerase
Glycopeptides	Vancomycin, teicoplanin	1953	1958	1960	Alteration of cell wall biosynthesis	Altered cell walls, efflux
Quinolones	Ciprofloxacin	1961	1968	1968	Alteration of DNA synthesis	Efflux, modification, target mutations
Streptogramins	Synercid, streptogramin B	1963	1998	1964	Alteration of cell wall biosynthesis	Enzymatic cleavage, modification, efflux
Oxazolidinones	Linezolid	1955	2000	2001	Alteration of formation of 70S ribosomal complex	Mutations in 23S rRNA genes followed by gene conversion
Lipopeptides	Daptomycin	1986	2003	1987	Depolarization of cell membrane	Modification of cell wall and cell membrane homeostasis
Diarylquinolines	Bedaquiline	1997	2012	2006	Inhibition of *Mycobacterial* ATP synthase	Mutation in *atpE* and drug efflux pumps

Abbreviations: YOD, year of discovery; YOI, year of introduction; YOR, year of observed resistance.

Today, antibiotics are regarded as “endangered species” as they are experiencing fast extinction because of the global occurrence of AR (Founou, Founou, & Essack, 2016). AR was noticed before the clinical application of antibiotics, and since then, antibiotic discovery, development, and use have been taking place alongside AR development (Abraham & Chain, 1988). Numerous strategies are evolved by bacteria to resist the toxic effects of most antibiotics that have been discovered and introduced into the medical and agricultural milieu, and this challenge has persisted especially with the emergence of multidrug resistance and superbugs, thus ushering us into the postantibiotic era (Alanis, 2005). This problem is not new as it has been predicted from the start of the antibiotic period as affirmed by Alexander Fleming's Nobel Prize speech in 1945 which reads as follows:

But I would like to sound one note of warning the time may come when penicillin can be bought by anyone in the shops. Then there is the danger that the ignorant man may easily underdose himself and by exposing his microbes to non‐lethal quantities of the drug and make them resistant. (Fleming, 1945)

It is a matter of concern now, because historically when pathogens resist an antibiotic, an alternative will be used, but recently, pan‐resistant organisms without new drugs to overcome them are rapidly increasing with specific concern are the “ESKAPE” pathogens, which include *Escherichia coli*, *Staphylococcus aureus*, *Enterobacter cloacae*, *Klebsiella pneumoniae*, *Pseudomonas aeruginosa*, and *Acinetobacter baumanii* as well as members of Enterobacteriaceae (MacGowan & Macnaughton, 2017).

## 3 THE DYNAMICS OF ANTIBIOTIC RESISTANCE

### 3.1 Causes of antibiotic resistance

#### 3.1.1 Overuse of antibiotics

Antibiotics have been traditionally utilized for the treatment of diseases caused by bacteria in important fauna, flora, and ornamental plants, and the use of oxytetracycline and streptomycin for this purpose is still rampant (Kumar & Pal, 2018). In the United States (US), for instance, streptomycin is sprayed on surfaces of plants like apples, pears, and ornamental plants for the control of the fire blight caused by the bacterium *Erwinia amylovora* (McManus & Stockwell, 2001)*.* Universally, the market consumption rate of antibiotics is assessed to range from 0.1 to 0.2 million tons yearly (Wise, 2002). In 2010, about 63,151 tons of antibiotics were used by livestock alone globally, and this usage is predicted to rise by 67% by 2030 if this issue is not addressed (Van Boeckel et al., 2015). Antibiotics are extensively used in medical, veterinary, and agricultural platforms for therapeutic, prophylactic, metaphylactic, and growth‐promoting purposes, with Beta‐lactam antibiotics like penicillins, cephalosporins, and carbapenems being the largest group of antibiotics consumed (50%–70%) by humans (Kumar & Pal, 2018). In contrast to our general expectations, it is essential to know that the hospital is not the only source of antimicrobials present in the environment community sewage (Kümmerer, 2008; Schuster, Hädrich, & Kümmerer, 2008). Notably, antibiotics are used to increase the protein content and reduce the fat content of meat during breeding (Gaskins, Collier, & Anderson, 2002). Antibiotics are also used for bee‐keeping and fruit production. Epidemiological studies suggest the existence of a link between antibiotic use and the evolution of antibiotic resistance. Therefore, the over‐consumption of antibiotics further initiates the development of antibiotic resistance (CDC, 2013; Read & Woods, 2014). Usually, when antibiotics are consumed in excess, they remove drug‐sensitive bacteria, and resistant bacteria are left to proliferate through natural selection (Read & Woods, 2014). Antibiotics are overprescribed worldwide despite the warnings regarding its overuse, and lack of regulation promotes the overuse of antibiotics by increasing their availability, accessibility, and affordability (Michael, Dominey‐howes, Labbate, Maria, & Elisabeth, 2014; The Antibiotic Alarm, 2013).

#### 3.1.2 Incorrect antibiotic prescriptions

Incorrect prescriptions of antibiotics may also lead to antibiotic resistance in bacteria (CDC, 2013). Studies have revealed that in about 30%–50% of clinical cases and treatment regimen, the choice of antibiotic and period of antibiotic therapy is not correct (CDC, 2013; Luyt, Bréchot, Trouillet, & Chastre, 2014). Antibiotics that are incorrectly prescribed often lead to problematic therapeutic effects and may pose potential clinical complications to patients (Lushniak, 2014). The concentration of subtherapeutic antibiotics may lead to the occurrence of AR by promoting gene modifications such as mutagenesis, deviations in gene expression, and horizontal gene transfer (HGT) (Viswanathan, 2014). Alterations in gene expression caused by antibiotics may trigger virulence, whereas HGT and increased mutagenesis may promote the occurrence of antibiotic resistance (Viswanathan, 2014). Studies have shown that piperacillin and/or tazobactam at a sublethal concentration induces wide‐ranging proteomic mutations in *Bacteroides fragilis* (Viswanathan, 2014).

#### 3.1.3 Extensive antibiotic use in agriculture/veterinary sectors

In both developing and developed countries, antimicrobials are generally used to boost the yield of livestock (Bartlett, Gilbert, & Spellberg, 2013; CDC, 2013; The Antibiotic Alarm, 2013). Antimicrobials are also believed to advance animal health and produce quality animal products (Michael et al., 2014). Unfortunately, these agents are eventually ingested by humans through residues in animal meat, which then suppress susceptible bacteria and allow resistant strains to thrive (Golkar, Bagasra, & Pace, 2014). Certain factors such as drug pharmacokinetics and physicochemical characteristics and the animal's biological processes influence the occurrence of antibiotic residues in animal products, which is highly correlated to the inefficient usage of the drugs. This drives antibiotic resistance even though they occur in small quantities and are not generally toxic. However, there is a dearth of information concerning the extent of occurrence of Veterinary Antimicrobial Agents (VAMAs) residues in animals globally (Beyene, 2015). Moreover, the spread of ARB to humans via animal products has existed for over 35 years, especially where farmers harbor similar ARGs with that found in farm animal's intestinal flora (Bartlett et al., 2013).

Research has demonstrated that antibiotic use in agriculture also affects the environmental microbiome (Bartlett et al., 2013; CDC, 2013). Approximately, 90% of the antibiotics dispensed on livestock are passed out through feces or urine and dispersed into manure, groundwater, and surface runoff (Bartlett et al., 2013; CDC, 2013). Recently, the contamination of several environmental sectors by VAMAs has been reported, which includes the soil (Li et al., 2015; Sun, Zeng, Tsang, Zhu, & Li, 2017; Wei, Ge, et al., 2016; Yang et al., 2016), plants (Bartrons & Peñuelas, 2017; Boonsaner & Hawker, 2015; Hussain, Naeem, Chaudhry, & Iqbal, 2016), water (Chen, Lang, Liu, Jin, & Yan, 2018; Ding et al., 2017; Dong, Zhang, Liu, Guo, & Hua, 2016; Li, Ho, Ying, & Deng, 2017; Zhang, Zhang, et al., 2018), and air (McEachran et al., 2015). VAMAs are capable of enhancing the selection of ARB within the environment, consequently causing serious ecological, plant, and human health problems, even at low concentrations (Kuppusamy et al., 2018). In the western and southern United States, tetracyclines and streptomycin are used as pesticides and sprayed on fruit trees (Golkar et al., 2014). This exposes environmental microorganisms to inhibitory agents, thereby altering the environmental ecology due to antibiotic resistance selective pressure (Golkar et al., 2014; Iwu & Okoh, 2019). The major groups of antibiotics utilized in animal husbandry are represented in Table 2.

**TABLE 2:** Major types of antibiotics used in animal husbandry (Source: Kemper, 2008; Tasho & Cho, 2016)

Antibiotic class	Members
β‐lactams	Benzylpenicillin, Amoxicillin, Cloxacillin, Dicloxacillin, Oxacillin, and Ampicillin
Peptides	Virginiamycin
Aminoglycosides	Spectinomycin, Kanamycin, and Apramycin
Cephalosporines	Cefquinom and Ceftiofur
Lincosamides	Lincomycin
Sulfonamides	Sulfapyridine, Sulfadimidine, and Sulfadimethoxine
Peptidomimetics	Bacitracin
Fluoroquinolones	Marbofloxacin and Enrofloxacin
Macrolides	Tylosin, Spiramycin and Erythromycin
Tetracyclines	Tetracycline, Chlortetracycline, Doxycycline, and Oxytetracycline
Ionophores	Monensin
Trimethoprim	

The overall quantity of antibiotics used worldwide in agriculture is estimated to range between 63,000 and 240,000 tons, although this figure is not precise because of inadequate collection of data and surveillance of antibiotic resistance in certain countries. More than 50% of antibiotics that are used for human therapy are now being commercialized and utilized in animal farming in many countries of the world (O'Neill, 2015). Annually, the average global ingestion of antibiotics per kg of livestock produced including cattle, chicken, and pigs is 45, 148, and 172 mg/kg, respectively (Van Boeckel et al., 2015). It is also postulated that the worldwide ingestion of agricultural antibiotics will increase by 67% in 2030 as a consequence of the expected rise in the number of animals needed for food (Kuppusamy et al., 2018). Also, antibiotic consumption in Russia, Brazil, South Africa, and China is proposed to double by 2030 as the population is projected to increase by 13% (Kuppusamy et al., 2018).

In the United States, approximately 80% of antibiotics vended are used for animal production, basically as growth promoters and prophylaxis (Ventola, 2015). In 2012, the estimates of the US Food and Drug Administration (FDA) disclosed that about 14,600 tons of antibiotics meant for animal production were sold as compared to just about 3,290 tons of antibiotics commercialized for human therapy in 2011 (Teillant & Laxminarayan, 2015). Also, China is regarded as the major producer of meat worldwide, and approximately 50% of the 210,000 tons of antibiotics produced by the country were deployed for farm animal production in 2015 (Collignon & Voss, 2015). In the same vein, 308, 932, 16, and 6 tons of VAMAs are expended for animal production in UK, Australia, Sweden, and Norway, respectively (Kim et al., 2011). In Brazil, Germany, and India, 5,600, 1,900, and 1,890 tons of antibiotics are utilized during animal production, respectively (Gelband et al., 2015). In Denmark, 11 tons of VAMAs are used in cattle production, 93 tons in pig production, and 0.4 tons in poultry production (DANMAP, 2015). In South Korea, 1,278 tons of VAMAs are used in animal production annually, and out of this, 112, 831, and 335 tons are used in cattle, piggery, and poultry farming, respectively (KFDA, 2006). With regard to the usage of antibiotics in animal husbandry, it is affirmed that they are mostly used in piggery followed by poultry and then cattle production (Kim et al., 2011), with tetracyclines, macrolides, and sulfonamides being the three most used antibiotics in veterinary settings (Du & Liu, 2012).

#### 3.1.4 Regulatory barriers

There have been several difficulties in pursuing regulatory approval for the discovery and development of antimicrobials including variations in the requirements for clinical trial among countries, ineffective communication channels, and fluctuations in licensing, administrative, and regulatory rules (Piddock, 2012). The US FDA in the last 20 years made some changes in the clinical trial standards, which has particularly made the clinical trials of antibiotics challenging (Wright, 2014). In some cases, smallholding companies are making an effort to fill the gap left by large pharmaceutical companies in the development of antibiotics, but their efforts are futile due to financial constraints and complexities involved in clinical trials (Piddock, 2012). Other factors such as awareness, political will, insufficient funding, coordination, monitoring, poor legal and regulatory framework, and data and technical capacity pose serious challenges to the formulation and implementation of policies enacted by the National Action Plan on antimicrobial resistance, which is aligned to the Global Action Plan on antimicrobial resistance endorsed for all countries to curb the threat of antibiotic resistance (IACG, 2018; WHO, 2016).

#### 3.1.5 Availability of few new antibiotics

Regulatory and economic barriers have forced most pharmaceutical companies to reduce the production of new antibiotics (Bartlett et al., 2013). Out of the 18 largest pharmaceutical industries, 15 have abandoned their research regarding the antimicrobial aspect of pharmaceuticals. In the same vein, antibiotic research is gradually dwindling due to these barriers (Piddock, 2012). Also, most pharmaceutical companies consider antibiotic development and production as an unwise investment, because antibiotics are curative and therefore used for only a short period; hence, they do not incur so many profits compared to medications used for treating chronic diseases, such as cancer, diabetes, psychiatric disorders, and asthma (Golkar et al., 2014; Piddock, 2012; Wright, 2014). Most physicians do not prescribe new antibiotics immediately because of the fear of promoting antibiotic resistance. They rather prefer to hold on to these new agents as reserve and, at most, prescribe them during serious illnesses while prescribing antibiotics that have demonstrated relative efficacy (Golkar et al., 2014; Gould et al., 2013). This has reduced investment returns (Piddock, 2012) and still does not change the fact that resistance might not occur when new antibiotics are prescribed (Gould et al., 2013).

#### 3.1.6 Nonexposure to antibiotics

The major contributing factor to antibiotic resistance selection and accumulation is exposure to antibiotics; hence, most studies that deals with the dynamics of antibiotic resistance usually focus on the exposure to antibiotics (Baym et al., 2016; Fridman, Goldberg, Ronin, Shoresh, & Balaban, 2014; Laehnemann et al., 2014; Lee, Molla, Cantor, & Collins, 2010; Toprak et al., 2011; Zhang et al., 2011). However, it has been shown that antibiotic resistance can evolve, persist, accumulate, and disseminate in the absence of any form of exposure to antibiotics, which could affect the rate at which antibiotic‐resistant bacteria multiply within the environment (Andersson & Hughes, 2010). One of the key strategies with which bacteria survive antibiotics is by mutagenically altering the target sites of the agent such that it no longer influences their targets, and this capability is often acquired via HGT. Usually, this mutation and HGT rates vary between different bacterial strains (Bolotin & Hershberg, 2015; Lynch et al., 2016) and can also be influenced by variations in environmental factors. This, in turn, affects the rate at which antibiotic resistance is acquired, which sometimes is independent of exposure to antibiotics (Diard et al., 2017; Hershberg, 2015, 2017). The following have been identified as resistance‐conferring agents besides antimicrobials: (a) heavy metals; (b) biocides including surfactants and disinfectants; and (c) antibiotic resistance genes (Singer, Shaw, Rhodes, Hart, & Balcazar, 2016). Others include xenobiotics like solvents, for example, octanol, hexane, toluene (Fernandes, Ferreira, & Cabral, 2003; Ramos et al., 2002), and plant‐derived chemicals (Friedman, 2015).

### 3.2 Antibiotic resistance crisis

Public health organizations label the swift evolution of ARB as a “nightmare scenario that could pose catastrophic consequences” (Viswanathan, 2014). However, this could have a good turn in the nearest future due to the recent advances in the control of the development of antibiotic resistance. In 2013, the Centers for Disease Control (CDC) stated that “the human race is in the postantibiotic era,” and WHO, in 2014, cautioned that antibiotic resistance is becoming dreadful (Michael et al., 2014). Globally, the pandemic of drug‐resistant *Staphylococcus aureus* and *Enterococcus* spp. poses the largest risk among Gram‐positive pathogens (CDC, 2013; Rossolini et al., 2014). Vancomycin‐resistant *Enterococci* (VRE) and other antibiotic‐resistant respiratory pathogens, including *Mycobacterium tuberculosis* and *Streptococcus pneumoniae*, are becoming epidemic (Golkar et al., 2014). Multidrug‐resistant Gram‐negative bacteria are gradually exhibiting resistance to virtually all existing antibiotics, creating a scenario that is suggestive of the “pre‐antibiotic era” (CDC, 2013; Golkar et al., 2014; Rossolini et al., 2014) and affecting most fields of medical practice (Golkar et al., 2014). The prevalence of these pathogens, including the extended‐spectrum beta‐lactamase‐producing *Escherichia coli* and *Neisseria gonorrhoeae* in the community, is becoming alarming (Rossolini et al., 2014). In South Africa, for instance, the management of clinical infections as well as food safety is jeopardized due to the increasing resistance exhibited by Gram‐negative bacteria against antibiotics of last resort, including carbapenems, tigecycline, and colistin (Sekyere, 2016). In 2015, the US CDC released a list of 18 antibiotic‐resistant pathogens considered to be a significant risk and categorized them into urgent, serious, and concerning threats (CDC, 2015). Three of them that were categorized as urgent threats are drug‐resistant *Clostridium difficile* which accounts for over 15,000 deaths in the United States annually, Carbapenem‐resistant Enterobacteriaceae that resists the effect of almost all available antibiotics, causing 600 deaths annually, and drug‐resistant *Neisseria gonorrhoeae*. Twelve were categorized as serious threats and include multidrug‐resistant *Acinetobacter*, drug‐resistant *Campylobacter*, fluconazole‐resistant *Candida*, extended‐spectrum beta‐lactamase‐producing Enterobacteriaceae (ESBLs), VRE, multidrug‐resistant *Pseudomonas aeruginosa*, drug‐resistant nontyphoidal *Salmonella*, drug‐resistant *Salmonella* Typhimurium, drug‐resistant *Shigella*, methicillin‐resistant *Staphylococcus aureus* (MRSA), drug‐resistant *Streptococcus pneumoniae*, and drug‐resistant tuberculosis, all of which cause approximately 22,500 annual deaths in the United States. The remaining three categorized under the concerned threat include erythromycin‐resistant Group A *Streptococcus*, clindamycin‐resistant Group B *Streptococcus*, and vancomycin‐resistant *Staphylococcus aureus* (VRSA).

### 3.3 The concept of multidrug resistance and superbugs

In the VET05‐R document of the “Clinical and Laboratory Standards Institute” (CLSI), it is stated that there is no universally recognized definition of multidrug resistance (MDR) (CLSI, 2011). However, CLSI recommends that MDR should solely denote innate resistance properties after a phenotypic antibiotic susceptibility testing (AST) has been conducted (CLSI, 2011). Schwarz et al. (2010) suggested that MDR occurs if (a) resistance is observed for antibiotics belonging to three or more different antibiotic classes after AST is performed and (b) bacteria cells harbor three or more resistance determinants that code for the different resistance pattern observed after AST and molecular screening of the resistance genes has been carried out. An exception to these definitions is when one resistance gene or gene complex causes structural and/or functional resistance to several antibiotics (Schwarz et al., 2010).

The term “superbugs” refers to microorganisms with numerous mutations that cause significant resistance to most antibiotic classes prescribed for the treatment of infections they cause, consequently leading to high morbidity and mortality, reduction in therapeutic options for these microbes, and increase in treatment cost and extended hospital stay (Davies & Davies, 2010). The super resistant strains, in some cases, have also acquired virulence properties with improved transmissibility (Davies & Davies, 2010). In the past half‐century, a strong connection between the therapeutic and prophylactic use of antibiotics in humans and animals and the consequent evolution of MDR, especially in Gram‐negative pathogens, including *Klebsiella pneumoniae*, *Salmonella enterica* and *Escherichia coli*, have been found to exist (Davies & Davies, 2010). This is particularly true for the beta‐lactam antibiotics and their corresponding deactivating beta‐lactamases (Davies & Davies, 2010). Three MDR bacilli found to be mostly implicated in nosocomial infections are as follows: extended‐spectrum beta‐lactamase (ESBL) Gram‐negative bacilli, MRSA, and VRE (Dzidic and Bedekovic, 2003).

### 3.4 Molecular development and general mechanisms of antibiotic resistance

Development of antibiotic resistance occurs through stable genetic change, including mutation, transformation, conjugation, and/or transduction (Holmes et al., 2016). Antibiotic resistance development occurs through two pathways: intrinsic and acquired. Intrinsic resistance develops due to the evolutionary adaptation of bacteria to their environment through mutations of pre‐existing genes found in the chromosomes of bacteria, causing reduced antibiotic affinity to their targets (Ventola, 2015). During this evolutionary process, genetic errors are accrued by bacterial cells in their genes (plasmids or chromosomes), and through vertical gene transfer, they transfer these genes to progeny cells, causing intrinsic or innate resistance (Founou et al., 2016). Acquired resistance occurs through HGT between bacteria of different or same species via conjugation, transformation, or transduction (Hayek, Gyawali, & Ibrahim, 2015). It could also occur through the acquisition of mobile extrachromosomal DNA elements including transposons, integrons, and plasmids instead of mutation. The acquisition of the genes that code for Beta‐lactamase (the enzyme responsible for the cleavage of Beta‐lactam antibiotics, such as cephalosporins and penicillins), is an example of acquired resistance (Doyle et al., 2006). Transformation happens when bacteria pick up free DNA containing the resistance genes from the environment; transduction occurs when DNA carrying the resistance genes is transferred via bacteriophages, which are viruses that affect bacteria; and conjugation takes place when the genes are transferred via conjugative pili (Roca et al., 2015).

With the regular identification of genes and vectors that code for antibiotic resistance, antibiotic resistance mechanisms including alteration of cellular antibiotic targets, prevention of antibiotic entrance into the cell, removal of antibiotics from the cells, degradation of antibiotics, and overexpression of membrane transporters are also being described. Antibiotics react with targets which are functional proteins such as ribosomal proteins and enzymes (Blair, Webber, Baylay, Ogbolu, & Piddock, 2014). A specific interaction occurs between an antibiotic and its target site (Blair et al., 2014). Antibiotics can be rendered ineffective when these target sites are altered through mutation (Blair et al., 2014). Also, deviations in the penetrability of the porins in the outer cell membrane render antibiotics ineffective as a result of the decreased influx of drugs (MacLean, Hall, Perron, & Buckling, 2010). Certain bacteria also have certain proteins such as the efflux pumps or plasma translocases which they use to expel a considerable amount of drugs out of their cells thereby reducing drug accumulation (Kumarasamy et al., 2010). Antibiotics can also be inactivated by enzymatic cleavage or by chemical modification (Liu, Constantinides, & Li, 2014). The use of an alternative metabolic pathway is also a way bacteria develop drug resistance (Liu et al., 2014). Through this means, substrates are utilized from the immediate environment to bypass the effects of antibiotics (Blair et al., 2014).

## 4 ANTIBIOTIC RESISTANCE IN THE AGRO‐ECOSYSTEM

### 4.1 Antibiotic resistance within and beyond various sections of the agricultural ecosystem

#### 4.1.1 Aquaculture

Aquaculture is regarded as the world's fastest developing food industry (Cabello, 2006; Miranda, Kehrenberg, Ulep, Schwarz, & Roberts, 2003). In 2015, the total global production of aquaculture was 76.6 million tons minus nonfood products and aquatic plants. Currently, it supplies about half of all seafood, which equates about 8% of the total food proteins of animal origin worldwide. The highest producers of aquaculture in the world include China (47.6 million tons), India (5.2 million tons), Indonesia (4.3 million tons), Vietnam (3.4 million tons), Bangladesh (2.1 million tons), Norway (1.4 million tons), Egypt (1.2 million tons), Chile (1 million tons), Myanmar (1 million tons), and Thailand (0.9 million tons) (FAO, 2017). Aquaculture is considered as “genetic reactors” or “hotspots for antibiotic resistance genes” where genetic recombination and exchange can take place, leading to the development of resistant strains of bacteria (Baquero, Martínez, & Cantón, 2008; Muziasari et al., 2016). Aquatic animals are increasingly becoming significant because they harbor deposits of antibiotics within their eatable tissues (Santos & Ramos, 2016).

Certain drugs and metal‐containing products such as copper are utilized in aquaculture to feed fishes, treat them, and thwart the fouling of the aquaculture (Burridge, Weis, Cabello, Pizarro, & Bostick, 2010). Examples of antibiotics allowed for aquaculture practices in the European countries include florfenicol, sulfonamides, sarafloxacin, oxytetracycline, and erythromycin (Santos & Ramos, 2016). Indeed, 70%–80% of some of these drugs are released into the water (Burridge et al., 2010; Cabello, 2006; Rigos, Nengas, Alexis, & Troisi, 2004), which alters the microbial populations present by selecting for the resistant strains of the environmental microbiota (Huerta et al., 2013). A low but substantial quantity of tetracycline (oxy‐ and 4‐epioxytetracycline), sulfonamide (sulfadimethoxine/ormetoprim), and macrolide (virginiamycin) were estimated in farm samples of tilapia (*Oreochromis* spp.), salmon, and trout (*Oncorhynchus* spp.) collected from 11 countries including Canada, Scotland, Mexico, Thailand, and China (Done & Halden, 2015). Although the concentration of antibiotics measured aligned with the specifications required by the US FDA, it was suggested that the selection and evolution of antibiotic resistance could be encouraged by the mere presence of these agents (Done & Halden, 2015).

Moreover, in another study in Shanghai City, shrimps and finfish samples were screened for 20 commonly used antibiotics belonging to the following classes: fluoroquinolones, Beta‐lactams, phenicols, tetracyclines, macrolides, and sulfonamides (Wang et al., 2017). The remnants of these antibiotics were recovered in 52% of the samples involving 40%–91% finfish samples and 17% shrimp samples. This study estimated that the total variance of exposure of men and women to antibiotics in the course of consumption accounted for 75% and 70%, respectively (Wang et al., 2017). Since over 10% of the sampled aquatic species contained antibiotics that exceeded the maximum residue limits (MRL), it is therefore certain that aquatic species can select for antibiotic resistance, thereby posing a serious threat to public health, especially in countries where MRLs are not severely imposed (Wang et al., 2017).

**FIGURE 1:**
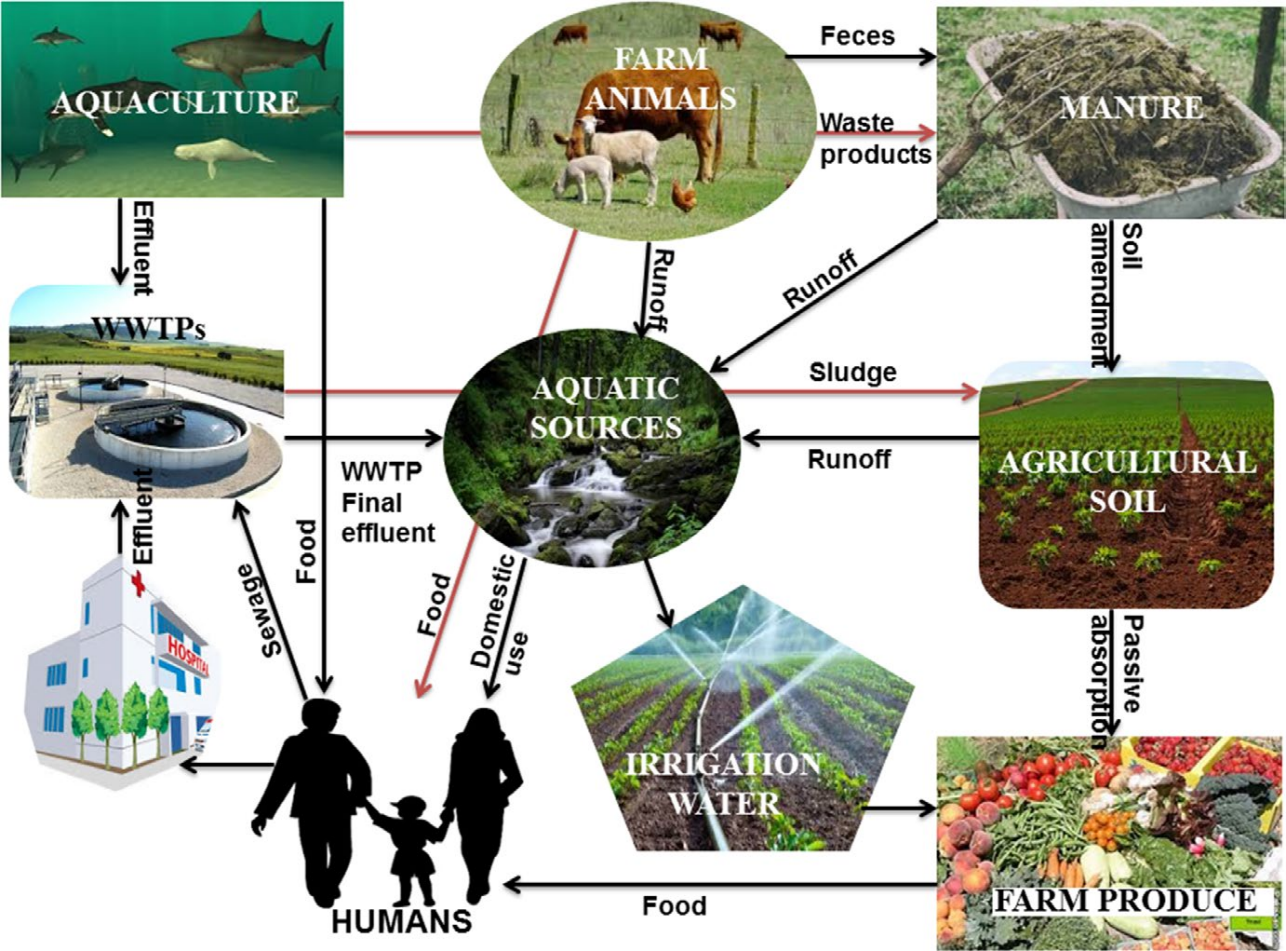
The possible transmission routes of antimicrobial agents, antibiotic resistance genes, and antibiotic‐resistant bacteria between the nexus of the agro‐ecosystem and finally to humans. A large proportion of antibiotics dispensed in medical, aquaculture, and animal farming are excreted through feces and urine by humans, fishes, and animals in a partially broken or unaltered chemical form and discharged into wastewater, sludge, and manure. These agents and naturally selected antibiotic‐resistant strains are then transferred to other sections of the agro‐ecosystem such as surface water bodies, irrigation water and agricultural soil, and eventually end up in food crops destined for both human and animal consumption, causing grave health implications. The movement of antimicrobial agents, ARGs, and ARB is depicted using line arrows. WWTPs, wastewater treatment plants

Seiler and Berendonk (2012) also suggested that the discharge of biocides and heavy metals into the natural environment via aquaculture activities also drives the selection and dissemination of AR within the marine environment. An example is seen where agents made up of copper are used to control parasites and the foul smell of a fish farm (Schlenk, Gollon, & Griffin, 1998). Some farms even use materials made up of copper alloys to build their cages (Burridge et al., 2010). In some commercially available fish feeds, high amounts of mercury (Choi & Cech, 1998), lead, iron, and cadmium (Kundu et al., 2017) have been detected. In a particular study, drug‐resistant strains of *Aeromonas* were isolated from fish and eel aquaculture systems (Penders & Stobberingh, 2008), harboring multiple integrons, gene cassettes, and plasmids for antibiotic resistance (Jacobs & Chenia, 2007). In some cases, the genes that confer resistance may be transferred horizontally to environmental, human, and veterinary pathogens (Cabello, 2006; Miranda et al., 2003). It is shown that aquaculture sludge increases the amount and dissemination of ARGs in the soil system and onto food crops (Wellington et al., 2013) when they are applied to agricultural soil, as illustrated in Figure 1.

#### 4.1.2 Animal farming

A complex antibiotic resistance transmission route exists between livestock and humans, thus posing health risks to both animals and humans (Marshall & Levy, 2011). Antimicrobials are dispensed to animals commonly used as food (e.g., poultry cattle and swine), for therapeutic, metaphylactic, and prophylactic reasons, and to improve the utilization of feeds and weight gain (Gustafson & Bowen, 1997). Depending on the pharmacokinetics of the drugs and the ability of the animal to transform the drugs, they are usually not completely broken down, and the bulk of the antibiotics is excreted in urine and feces as parent compounds or their metabolites, which in most cases are still very active (Kim et al., 2016, 2017; Sarmah, Meyer, & Boxall, 2006). The excretion rate of VAMAs, which as demonstrated in Table 3, is contingent on the kind and dosage of the antibiotic as well as the age of the animal. This is shown in a study where more than 72% of tetracycline administered orally was retained in manure unaltered after two days of administration (Winckler & Grafe, 2001). In another study, more than 90% of fluoroquinolones in the parent form were passed out by pigs after the drug was orally administered (Sukul, Lamshöft, Kusari, Zühlke, & &Spiteller, 2009). Consequently, these antimicrobials are then introduced into the agro‐ecosystem via soil amendment with animal feces containing antibiotics (after or before composting), bio‐solid, sewage sludge or sediment, irrigation with surface, waste or groundwater containing antimicrobials (Du & Liu, 2012)—as shown in Figure 1, or aerial movement in plants (McEachran et al., 2015). Pathogenic and commensal microbes present in the milieu can be transferred to humans, directly via contacts and food web or indirectly via farm effluents that are forced to evolve resistance strategies (Liebana et al., 2013).

**TABLE 3:** The uses of common VAMAs and their excretion rates

Antibiotic class	VAMAs	Use (reference)	Excretion rate (%) (reference)
Β‐lactam	Ampicillin	Remediation of infections caused by bacteria in animals and humans and to improve animal yield (Santos & Ramos, 2016)	60 (Hirsch et al., 1999)
	Amoxicillin	Remediation of infections caused by bacteria in animals and humans and to improve animal yield (Santos & Ramos, 2016)	90 (Park & Choi, 2008)
	Penicillin	Prevention and treating of infection, growth promoter (Webb & Fontenot, 1975)	NG
Tetracycline	Tetracycline	Used as prophylaxis in the control of human and animal infections caused by bacteria. They are also used to promote animal yield (Santos & Ramos, 2016)	72 (Winckler & Grafe, 2001)
Tetracycline	Chlortetracycline	Animal therapy, growth promoter (Kang et al., 2013; Kumar et al., 2005; Kumar, Thompson, Singh, Chander, & Gupta, 2004; Webb & Fontenot, 1975)	65 (Arikan et al., 2009)
	Oxytetracycline	Animal therapy, growth promoter (De Liguoro, Cibin, Capolongo, Halling‐Sørensen, & Montesissa, 2003; Webb & Fontenot, 1975)	21 (Montforts, 1999)
Sulfonamide	Sulfamethazine	Treatment of disease (Kang et al., 2013; Kumar et al., 2004, 2005; Seo, Cho, Kang, Jeong, & Jung, 2010)	90 (Halling‐Sørensen et al., 2001)
	Sulfamethoxine	Used as prophylaxis and therapeutics in humans, animals and fishes, as well as growth promoters in animals (Santos & Ramos, 2016)	15 (Jjemba, 2002)
	Streptomycin	Used as prophylaxis and therapeutics in humans, animals, and fishes, as well as growth promoters in animals (Santos &Ramos, 2016)	66 (Jjemba, 2002)
	Difloxacin	Used for prophylactic and therapeutic purposes in humans, animals, and fishes, as well as growth promoters in animals (Santos & Ramos, 2016)	90 (Sukul et al., 2009)
Macrolides	Erythromycin	Used in veterinary medicine to treat respiratory tract infections as well as used as growth promoters (Santos & Ramos, 2016)	5–10 (McArdell, Molnar, Suter, & Giger, 2003)
	Ivermectin	Used in veterinary medicine to treat respiratory tract infections as well as used as growth promoters (Santos & Ramos, 2016)	40–75 (Jjemba, 2002)
	Tylosin	Treatment of disease, growth promoter (De Liguoro et al., 2003; Kang et al., 2013)	28–76 (Halling‐Sørensen, Jensen, Tjørnelund, & Montforts, 2001)
Peptide	Virginiamycin	Growth promoter in poultry (Kang et al., 2013)	0–31 (Jjemba, 2002)
Aminoglycosides	Neomycin	Control and treatment and of bacterial enteritis. (De Liguoro et al., 2003; EMA, 2017; Webb & Fontenot, 1975)	NG
Ionophore	Monensin	Growth promoter especially in sheep and cattle (Kang et al., 2013; Webb & Fontenot, 1975)	NG
Peptidomimetics	Bacitracin	Growth promoter in poultry (De Liguoro et al., 2003; Webb & Fontenot, 1975)	NG

Abbreviation: NG, Not given.

Research has demonstrated that ARB can be transferred from animal farming to the soil and other environmental sites, including irrigation water and crops via manure. From the environment, it moves to the municipality facilities and probably ends up in the hospital (Heuer, Schmitt, & Smalla, 2011a). Hence, the following measures need to be put in place to reduce the development and spread of ARGs and ARB within the livestock settings: (a) Antibiotic used as growth promoters and other nonclinical application should be completely banned; (b) bio‐safety, bio‐security, and other hygienic measures in the farm and along the food chain should be improved, and only antibiotics prescribed by qualified veterinarians should be dispensed during therapeutic operations; (c) educational campaigns to farmers, veterinarians, and food processors should be encouraged; and (d) surveillance schemes on the spread of antibiotic resistance from livestock to humans should be adapted (Roca et al., 2015).

#### 4.1.3 Manure

The veterinary use of antibiotics is considered to be a vital source of antibiotics in the environment, a factor that immensely encourages the dispersal of antibiotic resistance (Ezzariai et al., 2018). Manure of animal origin serves as a significant reservoir of ARGs due to the promiscuous use of antibiotics in the animal setting, and this consequently elevates the level of antibiotic resistance within agricultural soil particularly after the application of antibiotics‐ and ARGs‐rich animal manure, thus posing environmental and human health problems (Zhang et al., 2019). A considerable quantity of ARGs are present in the manure and deposited on agricultural soil during soil amendment (Ghosh & LaPara, 2007; Heuer, Schmitt, et al., 2011). Manure is an established “hot spot” of bacteria that harbors ARGs within their MGEs (Binh et al., 2008; Wolters, Kyselková, Krögerrecklenfort, Kreuzig, & Smalla, 2015). High concentrations of antibiotics ranging from 91 to 136 mg/kg dry matter of tetracyclines (An, Chen, Wei, & Gu, 2015) and sulfonamides (Martínez‐Carballo, González‐Barreiro, Scharf, & Gans, 2007) have been detected from manure, which is consequently discharged into other niches of the environment including the soil (Sarmah et al., 2006), sediment (Rico et al., 2014), and surface and groundwater (Hirsch, Ternes, Haberer, & Kratz, 1999).

Furthermore, certain heavy metals, particularly Cu and Zn that are considered as drivers of antibiotic resistance, have been recovered from manure (Lu et al., 2014; Wang, Dong, Yang, Toor, & Zhang, 2013), and this is because they are used in animal husbandry as food additives (Xiong et al., 2010). The rapid use of antibiotics has caused their constant release into wastewater treatment plants (WWTPs), which are not originally designed to treat materials of this nature, thus presenting limited efficacy in their elimination; hence, a good part of these agents are discharged into the aquatic milieu via WWTPs effluents or released onto agricultural soil via sludge (Ezzariai et al., 2018). Animal manure is preferred over chemical fertilizers to enhance the fertility of soil and nutrient cycling during the production of organic foods because of their cheap and safe disposal capabilities (Torsvik & Øvreås, 2002). Animal manure is normally sourced from “confined animal feeding operations” (Ji et al., 2012), where a huge quantity of manure good enough for a large hectare of farmlands can be obtained from. In this kind of setting, antibiotics are used to promote the growth of animals as well as for therapeutic reasons. The animals absorb only a little fragment of these agents, while the rest (30%–90%) is excreted into the manure as illustrated in Figure 1 (Heuer, Schmitt, et al., 2011; Heuer, Solehati, et al., 2011; Ji et al., 2012; Negreanu, Pasternak, Jurkevitch, & Cytryn, 2012).

Application of animal‐based manure on fields for a long time and the direct discharge of animal sewage via drainage systems may cause the transfer of antibiotic residues as well as ARGs from livestock into nearby aquatic sources such as rivers, streams, lakes, and agricultural soil environments, thus posing hazard to the health of the environment and humans (Fang et al., 2018). In one instance, it is seen that organic fertilizer contributed 20 mg/kg of tetracycline into the soil (McEwen & Fedorka‐Cray, 2002). Moreover, the studies of Peng, Wang, Zhou, and Lin (2015) disclosed that the long‐term spread of fresh manure and compost on arable soil increased the magnitude of tetracycline resistance genes within the soil with the dominant *tet*G genotype, sharing a strong homology to those identified in pathogenic bacteria. When soils are treated with manure, unbroken antibiotics may select for resistant bacterial strains in the soil (Wang, Qiao, Chen, Su, & Zhu, 2015). ARGs can also be shared between soil microbiota through HGT (Jechalke, Heuer, Siemens, Amelung, & Smalla, 2014). The use of manure on agrarian soil used for the cultivation of food crops can potentiate the dispersal of ARB and ARGs onto food crops intended for human or animal consumption (Gaze et al., 2013).

Fortunately, with recent technological advances such as aerobic composting and anaerobic digest, residues of antibiotics and ARGs are profoundly reduced from manure before being applied to the soil (Wei, Qian, Gu, Wang, & Duan, 2016). Aerobic composting is one of the widely recognized methods used during manure production because of its simplicity, efficiency, and economic‐feasibility (Arikan, Mulbry, & Rice, 2009; Wang et al., 2016). However, the elimination efficacies of antibiotics and ARGs from the manure vary with different treatment procedures, manure types, and sites in a few scenarios (Zhao, Dong, & Wang, 2010; Zhu et al., 2013). No doubt, the treatment of manure using the composting method is economical and environment‐friendly. However, the process is characterized by a high density of microbial flora, involving a series of thermophilic, mesophilic, and thermo‐tolerant microbes. Thus, it could potentially lead to the transfer of ARGs and ARB to the soil if the process is not adequately managed (Zhang, Gu, et al., 2018).

Composting is defined as the process associated with the aerobic degradation of organic materials such as food leftover, paper, leaves, and manure performed by microorganisms usually under controlled conditions (Scoton, Battistelle, Bezerra, & Akutsu, 2016). Different communities of microorganisms including bacteria, fungi, algae, protozoa, and Actinobacteria occur during composting, which reflects the evolution and performance of the process (Ezzariai et al., 2018). During composting process, manure is stabilized through biological degradation, a process that reduces the levels of persisting organic pollutants (Poulsen & Bester, 2010; Sadef, Poulsen, Habib, Iqbal, & Nizami, 2016) and antibiotics (Liu et al., 2015; Mitchell et al., 2015). Just like organic materials whose physicochemical status affects their degradation during the composting process, antibiotic persistence, and mobility during composting are also driven by their physicochemical properties.

So far, only a little information on the elimination strategies of antibiotics and ARGs in manure is available. Wu, Wei, Zheng, Zhao, and Zhong (2011) linked abiotic transformations like epimerization or dehydration to the elimination of tetracyclines, which are unstable due to their chemical structure, and the products of the transformation (including 4‐epichlortetracycline, 4‐epioxytetracycline, 4‐epitetracycline, and anhydrotetracycline demeclocycline) were quantified and their removal assessed. The report of Kim et al. (2012) was premised on the hypothesis that sorption processes increase the removal of chlortetracycline during manure composting due to the elevated levels of divalent cations present within the organic media and the possible formation of chelates complexes with organic substrates. Therefore, their findings suggested that the addition of sawdust during manure composting positively influenced the removal of chlortetracycline in the manure composting process. Ho, Zakaria, Latif, and Saari (2013)—during 40 days' broiler manure composting—hypothesized that humic substances help in the removal of antibiotics from manure through their sequestration within the organic and inorganic matrices, thus rendering them less extractable for various antibiotics. Chen, Yu, Michel, Wittum, and Morrison (2007) demonstrated a 7.3 log reduction in the distribution of erythromycin ARGs during composting of swine manure. Similarly, Wang et al. (2016) reported a partial removal of tetracyclines ARGs at 2.4 log copies/g dry matter. Selvam, Xu, Zhao, and Wong (2012) showed complete removal of sulfonamides, tetracyclines, and fluoroquinolones ARGs after 42‐day manure composting.

Most researchers in the quest of improving the removal efficacy of antimicrobials and ARGs from the manure are tending toward isolation of antibiotic degrading microbes, process optimization, and other cost‐effective materials (Cui, Wu, Zuo, & Chen, 2016; Duan et al., 2017). One of the strategies involved the incorporation of a bulking agent during composting of animal manure as this optimizes the carbon to nitrogen ratio and humidity of the manure, which in turn increases the compost temperature necessary for composting. It is thought that the incorporation of the bulking agent during manure composting enhances the degradation of antibiotics and prevents the spread of ARGs within the manure (Zhang et al., 2019). Because of this, recent studies on the fate of antibiotics and ARGs in manure during composting are conducted by incorporating a bulking agent (Chai et al., 2016; Selvam et al., 2013).

#### 4.1.4 Agricultural soil

Agricultural soil serves as a rich reservoir of AR, having great implications for food safety and public health. As a result of the diverse microbial community including microbes that produce antibiotics present in the soil, the soil is considered as a reservoir of ARB and ARGs, which results from natural or anthropocentric practices such as manure application (Su, Wei, Yan, Qiao, & Guan, 2014). Globally, several antibiotics in high concentrations of about 1,000 µg/kg have been recovered from manures and manure‐based soil (Ho, Zakaria, Latif, & Saari, 2014; Li et al., 2015; Martínez‐Carballo et al., 2007; Yang et al., 2016). In this context, it supposes that the soil can represent an “under‐recognized reservoir” for antibiotic resistance that has previously developed or yet to develop in disease‐causing bacteria and must be put into consideration when assessing the risk factors that contribute to the worldwide spread of antibiotic resistance (Gatica & Cytryn, 2013). Other important routes of transmission of ARB, ARGs, and antibiotics to the soil environment include feces and urine of grazing animals, use of sewage sludge, bio‐solids, and dung during soil amendment as well as the use of recycled water for irrigation purposes (Carvalho & Santos, 2016; Li, 2014; Massé et al., 2014). Although it has been proposed that composting can reduce the levels of antibiotics in dung before they are applied to soil, recent studies have shown that it is difficult to completely remove all antibiotics present in the manure (Cessna et al., 2011; Dolliver, Gupta, & Noll, 2008; Ray, Chen, Knowlton, Pruden, & Xia, 2017). This exerts discerning pressure on indigenous community of microorganisms in the soil as well as augments the ARB and ARGs present in the soil (Chen, Ray, Knowlton, Pruden, & Xia, 2018; Chen, Lang, et al., 2018).

Usually, once antimicrobials are discharged into the environment, their fate and behavior become influenced by not only their physicochemical properties such as water solubility, volatility, lipophilicity, chemical properties, sorption, and/or sequestration capacities but also climate conditions and soil characteristics such as pH, organic matter content, ionic strength, and cation exchange capacity (Albero, Tadeo, Escario, Miguel, & Pérez, 2018). Other factors include microbial alterations, photo dilapidation, leaching, surface runoff, and plant uptake (Kuppusamy et al., 2018).

Sorption is the main process that determines the mobility and dispersal of antimicrobials in the environment, which governs the availability of antibiotics for plant uptake as they have to be in an aqueous phase for swift uptake (Albero et al., 2018). Sorption of antibiotics to soil controls the uptake of antibiotics by plants as well as their mobility within the soil environment and is affected by some factors including soil properties, steric configuration, and amphoteric and amphiphilic properties of the antibiotics (Wang & Wang, 2015). For instance, the ability of tetracyclines to form complexes with cations that are doubly charged increases their ability to form strong bonds with soil particles (Hamscher, Sczesny, Höper, & Nau, 2002). Another study proposed that the sorption of VAMA like sulfonamides to soil particles can be negatively impacted by the existence of dissolved organic materials (Kulshrestha, Giese, & Aga, 2004). A good relationship between pH and sorption of sulfamethazine and sulfathiazole to loamy and sandy soils was also affirmed in the findings of Kurwadkar, Adams, Meyer, and Kolpin (2007). Kodešová et al. (2015) concluded that the sorption of ionizable veterinary antibiotics is greatly affected by pH.

Usually, after a long period, antibiotics present in the soil become diffused into micro‐ and/or nanoparticles. Therefore, their bio‐accessibility and bio‐availability decrease. This temporarily reduces their biological contact and uptake, leading to their sequestration. The existence of sequestered antibiotics in the soil—which are usually in a form that is not bio‐available—are usually extended even though the process of sequestration tends to reduce their toxicity. However, it is important to understand that the process of sequestration can revocably lead to the discharge of sequestered antibiotics in a state that is bio‐available, usually in subinhibitory concentrations, which can now be continually detected in the soil over an extended range of time (Kuppusamy et al., 2018).

Complete biodegradation or alteration of antibiotics discharged into the soil is not usually possible as has been proven in the findings of Kreuzig and Höltge (2005), who discovered that about 18% of sulfadiazine in dung‐fertilized soil was still extractable even after incubation for 100 days. While some antibiotics are photodegradable, a study by Junge et al. (2011) revealed that photodegradation of antibiotics in the soil is limited due to inadequate penetration of light and should not be considered as another option for the degradation of antibiotics in the soil. Leaching is the vertical percolation of materials into the groundwater that preferentially occurs in a particular flow path and plays more of a role with antibiotics that are hydrophilic and/or not tightly sorbed to the soil particles (Jechalke et al., 2014). Therefore, antibiotics that are hydrophobic and/or interacting strongly with the soil particles may not leach beyond the surface soil (Aust et al., 2010; Ostermann et al., 2013). Hamscher, Abu‐Quare, Sczesny, Höper, and Nau (2000) discovered that 9.5 μg/kg chlortetracycline was recovered in 10‐cm topsoil samples received from farms after the second day of animal slurry application and a decreased concentration of about 0.7 μg/kg in soil samples collected at a depth below 80 cm. An even worst situation was observed by Stoob, Singer, Mueller, Schwarzenbach, and Stamm (2007), who noticed a 0.5% runoff loss for sulfonamides.

Surface runoffs and transport facilitated by particles also increase the transfer of antibiotics in the soil to the broader environment (Larsbo et al., 2008; Popova, Bair, Tate, & Parikh, 2013). Stoob et al. (2007) noticed a 0.5% runoff loss for sulfonamides. Antibiotics in the soil can also be taken up by crops cultivated on that soil, although in small amounts (Azanu et al., 2016; Pan & Chu, 2017; Tasho & Cho, 2016; Wu, Dodgen, Conkle, & Gan, 2015; Wu, Huang, et al., 2015), but sufficient enough to prompt phytotoxic effects on the growth of plants (Bártíková, Podlipná, & Skálová, 2016; Pan & Chu, 2016). The uptake of antibiotics by plants depends on the species of the plant and the antibiotic (Du & Liu, 2012). It was observed in a study that 2–17 ng/g chlortetracycline was taken up in onion, corn, and cabbage but tylosin‐like antibiotics with bigger molecular weight were not (Kumar, Gupta, Baidoo, Chander, & Rosen, 2005). Moreover, 2.8–13 μg/kg and 6–170 μg/kg of levamisole, trimethoprim, and florfenicol were detected in lettuce and carrot, respectively, grown on sandy soil with the original concentration of 1 mg/kg (Boxall et al., 2006).

ARGs from the soil resistome can be dispersed into the food web through polluted groundwater or food crops (Figure 1) and eventually to human pathogens in some cases, affecting human health (Udikovic‐kolic, Wichmann, Broderick, & Handelsman, 2014). Some researchers have proofed that certain agricultural activities such as the application of animal dung on farm soils expand the rate of intrinsic resistance in soil (Knapp et al., 2010; Popowska et al., 2012). The presence of ARGs on the surface of vegetables and fruits has also been reported (Abriouel et al., 2008; Boehme, Werner, Klare, Reissbrodt, & Witte, 2004; Durso, Miller, & Wienhold, 2012; Rodriguez et al., 2006), which is related to the fact that the group of bacteria found on plant surfaces can be influenced by various antibiotic‐resistant strains found in the soil.

#### 4.1.5 Freshwater bodies

As the main receptacle of pollution from industrial, agricultural, aquaculture, and domestic settings, aquatic environments constitute a reservoir for the evolution of new AR (Bondarczuk, Markowicz, & Piotrowska‐Seget, 2016). Growing concern regarding the occurrence of ARB and ARGs in the aquatic milieu, especially in surface water bodies such as rivers, dams, and lakes, is established (Blaak et al., 2015). Hence, strategies for improving water quality are urgently required. Increase in the distribution of ARB, ARGs, and antibiotics in freshwater has frequently been allied to the discharge of effluents of WWTP as well as other factors including the increase in population density, industrial activities, and agricultural activities along with the courses of freshwater bodies (Sabri et al., 2018). In South Africa, Adefisoye and Okoh (2016) and Igwaran, Iweriebor, and Okoh (2018) detected antibiotic‐resistant pathogenic strains of *E. coli* harboring ARGs including *str*A, *aad*A (aminoglycosides); *cat*I, *cmlA*1 (phenicols); *bla*_TEM_ (beta‐lactamase); *tet*A, *tet*B, *tet*C, *tet*D, *tet*K, *tet*M (tetracyclines), and mcr‐1 (colistin); *erm*A (macrolide), respectively, in final effluents of WWTPs, which is to be discharged into freshwater bodies. Odjadjare and Okoh, (2010) also detected antibiotic‐resistant *Listeria* spp. harboring the *sul*II (sulfonamide) ARG in municipal wastewater effluents destined to be discharged in a freshwater body.

As presented in Figure 1, ARB possibly selected by the indiscriminate use of antibiotics are collected from various sources including hospital, rural, urban, municipal, or agricultural effluents, and mixed with resident species in the water bodies where these effluents are discharged (Balcázar, Subirats, & Borrego, 2015). This produces two effects with regard to the spread of antibiotic resistance: First, the water bodies that play a pivotal role in agricultural and domestic activities are contaminated with multidrug‐resistant strains of bacteria (Berendonk et al., 2015), and secondly, genetic exchange of ARGs between contaminating ARB and resident bacteria through phages or integrons occurs, thus altering the microbiome of the aquatic ecosystem (Xu et al., 2016). These transfers have been reported in several studies.

In India, the findings of Devarajan et al. (2016) demonstrated that beta‐lactamase genes (*bla*_SHV_ and *bla*_NDM_) were detected in the sediments of the Cauvery River contaminated by urban and hospital wastewaters. In Spain, Lekunberri, Villagrasa, Balcázar, and Borrego (2017) noticed that the concentrations of ARGs including *erm*B (macrolide), *qnrs* (fluoroquinolone), and *tet*W (tetracycline) were significantly different at the Ter River upstream and downstream discharge point of WWTP effluent. The Pearl River in China, which is profoundly predisposed to anthropological activities, was reported to contain tetracycline resistance genes including *tet*C, *tet*B, *tet*M*, tet*W*,* and *tet*O (Chen, Liang, Huang, Zhang, & Li, 2013). In Nigeria, Titilawo, Obi, and Okoh (2015) detected antibiotic‐resistant *E. coli* harboring the *sul*I, *sul*II (sulfonamide resistance genes), *amp*C, *bla*_TEM_, *bla*_z_, (beta‐lactamases), *tet*A, *tet*B, *tet*C, *tet*D, *tet*K, *tet*M (tetracycline resistance genes), *cat*I, *cat*II, *cm*IA1 (phenicol resistance genes) and *aac*C2, *aph*A1, *aph*A2, *aad*A, *str*A (aminoglycoside resistance genes) in rivers where anthropogenic activities such as fishing, animal rearing, tourism, car washing, swimming, and so on are carried out. Ling's , Yang, Huang, Zou, and Luan (2013) findings showed that *sul1* and *sul2* (sulfonamide resistance genes), *tet*A*, tet*G*, tet*X*, tet*O*, tet*M, and *tet*C (tetracycline resistance genes) were often detected in the Beijing River, which is located in the most urbanized region of China. The spread of ARB, ARGs, and antibiotics in freshwater occurs not only in the water and sediment but also in biofilms (Proia et al., 2016), and the guts of aquatic animals (Fu et al., 2017), which also affect the marine environment including marine water and sponge species (Chen et al., 2013; Hatosy & Martiny, 2015; Laport et al., 2016).

#### 4.1.6 Irrigation water

Water used for irrigation of crops could serve as an important potential route of human exposure to AR (O'Flaherty & Cummins, 2017) as it is established to be the foremost cause of farm produce contamination (EFSA BIOHAZ Panel, 2013; De Roever, 1999). Water used for irrigation may become polluted by either nonpoint pollution sources, such as agricultural runoffs, or direct contact with sewage and manure (De Roever, 1999). Residues of antibiotics are extensively spread in the environment, including numerous compartments of the aquatic milieu, such as municipal effluent, hospital effluent, surface water, and groundwater usually at amounts ranging in the nearest ng/L to a few µg/L (Azanu et al., 2016). Kolpin et al. (2002) detected about 110 ng/L tetracyclines in surface water, and Andreozzi et al. (2004) detected about 120 ng/L amoxicillin in WWTP effluent. As it has been shown that surface water bodies such as lake water and treated wastewater contain ARG (Czekalski, Berthold, Caucci, Egli, & Bürgmann, 2012), water obtained from these sources for irrigation of plants may transfer ARB and ARGs onto food crops. Groundwater used for irrigation of crops can be considered as a vital “hidden resource” (Araújo et al., 2017), which can be contaminated by human activities and can persist for a long time until the source of pollution is eliminated (European Commission, 2008).

The most important contamination route of antibiotic‐resistant pathogens on vegetables at a preharvest stage is through the irrigation water (Figure 1). This is validated by several researchers, who have detected antibiotic‐resistant disease‐causing bacteria on fruits and vegetables (Bezanson, Macinnis, Potter, & Hughes, 2008; Ruimy et al., 2010; Schwaiger, Helmke, Hölzel, & Bauer, 2011). In Belgium, 5%, 4.3%, 3.1%, 3.1%, and 3.1% of *E. coli* isolated from irrigation water sources (rain harvested and borehole water) used in eight lettuce farms were resistant to tetracycline, ampicillin, streptomycin, sulfonamides, and trimethoprim, respectively, with every possibility of being transferred to the lettuce crops, which usually undergoes minimal processing before consumption (Holvoet, Sampers, Callens, Dewulf, & Uyttendaele, 2013). Nontongana, Sibanda, Ngwenya, and Okoh (2014) detected antibiotic‐resistant pathogenic strains of *E. coli* in Kat river, South Africa, where water for irrigation of citrus orchard and Municipal waterworks is sourced. Iwu and Okoh, (2020) detected antibiotics resistant strains of *Listeria monocytogenes* from irrigation water sources in the Eastern Cape Province of South Africa.

In another research in France by Tamtam et al. (2011), the presence of antibiotics was tested for on farmland located about 65 km downstream of Paris that was irrigated for more than 100 years with wastewater and stopped for about four years as at the time of the experiment. The concentration of antibiotics including nalidixic acid (21–22 µg/kg), flumequine (6–7 µg/kg), and oxolinic acid (5–6 µg/kg) was observed on the soil site close to the irrigation water outlets. This suggests that these contaminants from irrigation water can persist for a long time in the soil and can either leach to the groundwater or be absorbed by plants. In the same vein, Chen et al. (2014) noticed that the soil irrigated with wastewater harbored more antibiotics and ARGs compared to the soil that is not irrigated at all.

Moreover, high levels of antibiotics including tetracyclines (145.2 mg/kg) and quinolones (79.2 mg/kg) were detected in the soil of public parks in China irrigated with WWTP effluents (Wang et al., 2014). In Colorado, USA, Kinney, Furlong, Werner, and Cahill (2006) also detected high levels of antibiotics including erythromycin (154–611 ng/L), sulfamethoxazole (3–59 ng/L), and trimethoprim (2–42 ng/L) in the recycled water used for irrigation as well as in the implicated soil with erythromycin, being the most highly detected antibiotic in the soil. In Israel,Negreanu et al. (2012) noticed that treated WWTP effluents and freshwater contributed about 4 × 10^5^ to 4 × 10^6^ tetracycline‐resistant bacteria and about 4 × 10^6^ to 4 × 10^7^ ciprofloxacin‐resistant bacteria, both of which are culturable per square meter of the soil daily. Sallach et al. (2015) detected sulfamethoxazole in lettuce cultivated in a glasshouse and irrigated with artificial wastewater at concentrations ranging between 84 and 822 ng/g fresh weight. This shows that irrigation water serves as a potential transmission route of antibiotics, ARBs, and ARGs to farm produce, thus posing a risk to human health.

#### 4.1.7 Crop production

Antibiotics, ARB, and ARGs are constantly deposited in agricultural lands (Zhang et al., 2017), and plant crops become exposed to them as a result of their tenacity in the agro‐ecosystem, depending on certain factors such as the compounds physicochemical properties, environmental conditions, and absorption capabilities (Azanu et al., 2016; Dolliver, Kumar, & Gupta, 2007). The application of manure, which is often referred to as a rich source of ARGs and antibiotics to soil, enhances the evolution of ARB within the soil microbiome, which in extension influences the bacterial communities of plants grown above and below the soil (Zhu, Chen, Chen, & Zhu, 2017). Therefore, the microbiome of plants cultivated on soil enriched by manure is subject to variations of the microbial community of the soil. In some studies, ARGs have been detected from the surfaces of fruits and vegetables. Wang et al. (2015) recovered ARGs from different parts of lettuce grown on soil amended by manure, including the phyllosphere, root, and leaf endophytes.

The direct use of antimicrobials on plants from both quantitative and qualitative aspect is generally minimal compared to their use in medical settings. However, antimicrobials sprayed over plants become discharged into water systems and soil, affecting human, animal, and environmental resistome (Thanner & Drissner, 2016). In some cases, antimicrobials in the soil are passively absorbed into plants via plant roots or through water transportation (Hu, Zhou, & Luo, 2010). It is shown that certain pharmaceuticals like amoxicillin, oxytetracycline, trimethoprim, sulfamethoxazole, ofloxacin, tylosin, tetracycline, sulfamethazine, and ciprofloxacin can be absorbed and accumulated by plant crops including rice, carrot, cabbage, corn, potato, spinach, cucumber, lettuce, tomato, and wheat from the growth media via their roots (Ahmed et al., 2015; Chowdhury, Langenkämper, & Grote, 2016; Franklin, Williams, Andrews, Woodward, & Watson, 2016; Herklotz, Gurung, Vanden Heuvel, & Kinney, 2010; Hussain et al., 2016; Kang et al., 2013; Pan, Wong, & Chu, 2014). Humans, and in some cases animals, consume these plant crops, consequently causing the development of antibiotic resistance. There is an increasing threat of antibiotic resistance pollution of food crops emanating from the complex nexus of the agro‐ecosystem (Figure 1) and its effects in the food chain.

Table 4 shows some selected studies on the occurrence of ARGs in some sections of the agro‐ecosystem.

**TABLE 4:** Some selected studies on the occurrence of antimicrobial resistance genes in sections of the agro‐ecosystem

Section of the agro‐ecosystem	Antibiotics	Predominant ARGs	Country	References
The effluent of coastal Aquaculture	Tetracyclines	*Tet*B, *tet*D	South Korea	Jang et al. (2018)
Swine feces	Ampicillin; tetracycline; aminoglycoside	*Amp*C; *tet*A; *str*A	South Africa	Iwu, Iweriebor, Obi, Basson, and Okoh (2016)
Dairy and swine manure	Tetracycline; aminoglycosides; sulfonamide; macrolide‐lincosamide‐streptogramin type B	*tet*(MQST); *aad*(A), *str*(AB); *sul1*; *erm*(ABCF)	London	Marti et al. (2013)
Dairy cattle farm	Beta‐lactams; tetracycline; aminoglycoside	*amp*C*, bla*_CMY_, *bla*_CTX‐M_, *bla*_TEM_; *tet*A; *str*A	South Africa	Iweriebor, Iwu, Obi, Nwodo, and Okoh (2015)
Agricultural Soils	Tetracycline	*tet*(X)	Russia	Danilova, Galitskaya, and Selivanovskaya (2017)
Agricultural Soils	Tetracycline; aminoglycosides; sulfonamide; beta‐lactams	*tet*A*, tet*B*, tet*C*; aadA, aac(3)‐IIa; sul*I*, sul*II*; Amp*C*, bla*_TEM_, *bla*_CTX‐M_ *group9*, *bla*_VEB_	South Africa	Iwu and Okoh (2020)
Soil	Tetracycline; aminoglycosides; erythromycin	*tet*(B), *tet*(D), *tet*(O), *tet*(T), tet(W);*str*(A), *str*(B),*aac*; *erm*(C), *erm*(V), *erm*(X), *msr*(A), *ole*(B), *vga*	Poland	Popowska et al. (2012)
River	Sulfonamide; tetracyclines.	S*ul1*;*tet*G, *tet*B, *tet*A, *tet*Q, *tet*M	China	Yang, Liu, Xu, Wei, and Wang (2017)
Freshwater bodies	Sulfonamide; beta‐lactam; tetracyclines; aminoglycosides	*dfr*1; *amp*C; *tet*A, *tet*E; *str*A	Nigeria	Adesiyan, Bisi‐Johnson, Ogunfowokan, and Okoh (2019)
Irrigation water	Tetracycline; aminoglycosides; sulfonamide; beta‐lactams	*tet*B*, tet*C*; aac(3)‐IIa; sul*I*, sul*II*; Amp*C*, bla*_TEM_, *bla*_CTX‐M_ *group9, bla*_VEB_	South Africa	Iwu and Okoh (2020)
Irrigation water and vegetables	Beta‐lactams; tetracyclines.	*bla*_TEM_;*tet*A, *tet*B	Portugal	Araújo et al. (2017)
Harvested vegetables	Tetracyclines; sulfonamide	*tet*A, *tet*AP, *tet*C, *tet*G, tetL*tet*BP, *tet*M, *tet*O, *tet*W,tetX; *sul*I, *sul*II	China	Wang et al. (2015)
Animal meat	Aminoglycoside; beta‐lactams; tetracyclines; sulfonamides	*aad*A; *Amp*C; *tet*A; *sul*I	South Africa	Jaja, Bhembe, Green, Oguttu, and Muchenje (2018)
Humans living in a livestock‐dense area	β‐lactams	*bla*_CTX‐M‐15_ *bla*_CTX‐M14/17_ *and bla*_CTX‐M‐1_	Netherlands	Wielders et al. (2017)

### 4.2 The background and baseline levels of antibiotic resistance in the agro‐ecosystem

The existence of antibiotics, ARB, or ARGs in the agro‐ecosystem is not solely correlated to anthropogenic activities since certain bacteria and fungi living freely in the environment can extrude certain compounds that are structurally similar to antibiotics and can encourage the emergence of ARB and ARGs within the soil and water milieu (Rothrock et al., 2016). Studies have shown that antibiotic resistance in the environment is an old phenomenon (Bhullar et al., 2012; Brown & Balkwill, 2009; Miteva, Sheridan, & Brenchley, 2004) and the recent elevated levels of ARGs noticed in the environment are attributed to anthropogenic activities (Heuer, Solehati, et al., 2011; Knapp et al., 2010; Pruden, Pei, Storteboom, & Carlson, 2006). This shows that it is almost impossible for any environmental niche to be considered pristine because of the global hydrologic cycle and long‐range airborne transport of particulate matter and microbes (Allen et al., 2010). It also shows that antibiotic resistance in the environment is of both natural and anthropogenic origin and does not validate the fact that ARB and ARGs detected in environmental samples where antibiotics were applied was actually due to the antibiotic. This, therefore, calls for the need to obtain information on the background (innate) and baseline (reference) levels of antibiotic resistance in the agro‐ecosystem before the application of antibiotics.

Rothrock et al. (2016) defined background levels of antibiotic resistance in the environment as the level of antibiotics, ARB, and ARGs in the environment not yet influenced by anthropogenic activities such as application of manure to agricultural soil; and baseline levels of antibiotic resistance as the numerical averages and ranges of the levels of antibiotics, ARB, and ARGs in the environment at the beginning of a study. Obtaining these data are imperative when carrying out antibiotic resistance surveillance in the agro‐ecosystem to (a) validate the causal effects of antibiotic resistance in the agro‐ecosystem, (b) accurately evaluate any significant changes that might occur in the levels of ARB or ARGs in the course of a study, and (c) establish the true link between anthropogenic application of antibiotics in agriculture and the spread of antibiotic resistance (Rothrock et al., 2016). This will go a long way in identifying the transmission routes of antibiotic resistance in the agro‐ecosystem thus curbing its spread to the food web, therefore, limiting the levels of risks posed to public health.

### 4.3 Methods used for detecting environmental antibiotic resistance

#### 4.3.1 Antibiotic susceptibility testing

Routine techniques in detecting antibiotic resistance are focused on the phenotypic evaluation of bacterial growth in the presence of a different set of antibiotics (March‐rosselló, 2017). Isolation of pure cultures of pathogens has always been a critical part of medical microbiology (Karkman, Do, Walsh, & Virta, 2018). Antibiotic susceptibility testing (AST) is used in clinical and research microbiology laboratories to identify drug‐resistant pathogens and their susceptibility pattern to commonly used antibiotics during the treatment regimen (Tang, Apisarnthanarak, & Hsu, 2014). This approach of detection encompasses the observation of the growth pattern of a specific amount of microbes inoculated in a growth media that contains a serial dilution of test antibiotics (Tang et al., 2014). The E‐test strips, disk susceptibility testing, automated systems, and broth dilution are the most commonly adopted methods for AST (March‐rosselló, 2017), which have been explicitly revised elsewhere (Jorgensen, Ferraro, Jorgensen, & Ferraro, 2009). These methods produce results that are either quantitative or qualitative (e.g., disk susceptibility testing), but whichever the case, they must be interpreted using standards that are frequently updated and published by expert bodies like the **“**European Committee on Antimicrobial Susceptibility Testing (EUCAST)” (EUCAST, 2014) or the “CLSI” (CLSI, 2014).

The drawback with the phenotypic susceptibility testing method is that it is not time effective and cannot describe the precise antibiotic resistance mechanism. Also, this method cannot be used on environmental bacteria that are viable but not culturable, hence poses innate culture bias (Franklin, Aga, et al., 2016). However, an accurate guess can be made based on the available test results and the microorganism species tested (Jorgensen et al., 2009). With the growing availability and cost‐effectiveness of next‐generation sequencing, whole‐genome sequencing is now recommended and utilized for the detection of antibiotic resistance, and the results have been excellent, especially in certain organisms like *Staphylococcus aureus* (Holden et al., 2013). Nonetheless, phenotypic methods, compared to whole‐genome sequencing and other molecular methods, remain more reliable as novel resistance mechanisms emerge frequently (Jorgensen et al., 2009).

#### 4.3.2 Molecular methods

##### Polymerase chain reaction

The polymerase chain reaction (PCR) is the most applied molecular procedure utilized for diagnostic purposes. This is because it does not only detect pathogens but the resistance and virulence gene determinants of the pathogen. It takes approximately 12 hr for a conventional PCR to perform, and this is partitioned into three important steps. The first step is DNA extraction, which is followed by the second step consisting of DNA amplification in a thermocycler, and then the detection of amplified DNA materials in gel electrophoresis (Rosselló & Pérez, 2016). A real‐time PCR was manufactured—to reduce this time—because it amplifies and detects amplified materials simultaneously. Types of real‐time PCR that identify pathogens and screen resistance determinants commonly marketed include Verigene® system (Nanosphere), which confirms up to five species and four genera of Gram‐negative bacteria as well as ARGs such as ESBLs (CTX‐M) and carbapenems (VIM, OXA, IMP, KPC) with specificity of up to 93% (Ledeboer et al., 2015); the Film Array Blood Culture Identification Panel system (Biofibre Diagnostics), which detects up to 11 species and 15 genera of Gram‐positive bacteria as well as ARGs such as *van*A/B, *KPC,* and *mec*A with up to 99% specificity (Pence, McElvania TeKippe, & Burnham, 2013); and GeneXpert^®^ system, which performs real‐time PCR in single‐use disposable cartridges.

The quantitative PCR (qPCR) is used for the analysis of ARGs in DNA found in the environment without culturing. The set back with this method is that you need to extract the DNA of so many different bacteria from different environmental sample matrices to be able to compare (Franklin, Aga, et al., 2016). Also, you must have prior knowledge of the primer design of known gene or genes with similar homology to known ones. However, these limitations can be addressed by using a high‐throughput qPCR (Looft et al., 2012; Stedtfeld et al., 2008; Zhu et al., 2013). qPCR assays can simultaneously quantify hundreds of ARGs as parallel assays in one run, hence creating a chance for the quantification of genes of interest, sequences that are linked to MGEs as well as the quantification of genes that are specific to certain bacteria species in the environment (Karkman et al., 2018). Another way of addressing such limitations is the use of the droplet digital PCR (ddPCR). The ddPCR is a new technology that quantifies nucleic acids present in samples, with high sensitivity and precision. Unlike the qPCR, the ddPCR partitions the PCR reaction into thousands of individual droplets before amplification, such that the quantification of the nucleic acid occurs independently within each droplet (Martinez‐Hernandez et al., 2019). This offers an advantage of absolutely quantifying nucleic acids in samples without standard curves. It produces data that are more reproducible even in the presence of contaminants that can potentially inhibit the Taq polymerase and/or primer annealing (Taylor, Laperriere, & Germain, 2017).

##### Metagenomics

Metagenomics captures the sequences of the DNA of a whole community and can be used without having any prior knowledge of the target resistance genes. It has been applied in various environments to detect ARGs and capture the entire resistome of the environment as it is not restricted to only a few genes of choice (Karkman et al., 2018). In metagenomics, delineation of detected ARGs relies on confirmed genes that are present in public ARGs databases (Gibson, Forsberg, & Dantas, 2015; Gupta et al., 2014; Jia et al., 2017; Lakin et al., 2017; Liu & Pop, 2009; Zankari et al., 2012). The ones that have been experimentally confirmed are the most reliable (Wallace, Port, Smith, & Faustman, 2017), such as the hidden Markov model‐based database, Resfams (Gibson et al., 2015), and a recent version of CARD database (Jia et al., 2017). Because of the scarcity in the number of ARGs present in the environment compared to other functional genes, deep sequencing is usually required to capture the whole assortments of ARGs present (Bengtsson‐Palme et al., 2016; Yang, Li, Ju, & Zhang, 2013). Most platforms for metagenomics sequencing usually generate short reads that provide only limited data about the genes sequenced. However, assembling of short reads into longer, overlapping DNA segments, called contigs, may be able to provide evidence about the genetic location of the genes and the phylogeny of the genes. Reconstruction of partial or complete genomes can be done from metagenome data (Albertsen et al., 2013; Hultman et al., 2015), and this knowledge is necessary for assessing the level of risks associated with the presence of ARGs in the environment.

##### Functional metagenomics

Functional metagenomics deals with the cloning and expression of environmentally sourced DNA in a host in the laboratory. This technique has more advantage than the PCR and metagenomics because it requires no prior knowledge of the ARGs. In this technique, DNA from the environment is cloned usually in large fragments (10–200 kb) in a laboratory host, such as *E. coli*, after which the susceptibility of the host to various antibiotics is assessed. Clones that exhibit phenotypic resistance are further screened for resistance genes using silicon analysis, mutagenesis, or subcloning. The set back of this procedure is that it is arduous and time‐consuming because it is challenging to clone and express genes in a given host. A combination of proteomics and functional metagenomics will help reduce the hitches of screening potential clones that contain each DNA segments by identifying the expressed proteins in a high‐throughput manner and comparing it to a confirmed strain without having to identify the cloned DNA and alleged new resistance determinants (Fouhy, Stanton, Cotter, Hill, & Walsh, 2015). Generally, the molecular methods offer certain advantages compared to the culture‐based methods such as avoiding culture bias, offer better means of source tracking ARB/ARGs within the agricultural systems, and provide more information from the extracted DNA or RNA or proteins present in environmental samples (Franklin, Aga, et al., 2016), hence highly recommended when carrying out the surveillance of antibiotic resistance in the environment.

#### 4.3.3 Other methods

##### Microarrays

In this method, target molecules that are hybridized to a particular probe immobilized on a solid base are detected using image analysis (March‐rosselló, 2017). Microarrays can be utilized to detect an ample quantity of resistance genes in one assay so long as the probes—in this case, oligonucleotides—are fixed very close to each other. Several microarrays that can detect numerous resistance genes including ESBLs, AMPCs, and carbapenemases are available in the market, such as Check‐MDR CT102, Check‐MD CT103, and Check‐MDR CT103 XL (Checkpoints Health BV).

##### Immuno‐chromatographic methods

This technique is used to detect bacterial enzymes that hydrolyze antibiotics. In this method, the test bacteria are suspended in a diluent, and few drops of this diluent are transferred onto one end of a nitrocellulose strip. The bacteria migrate to the other end of the strip via capillarity, and if positive, a clear band is shown on the test region of the strip where antigen‐specific to the antibody of the bacterium is attached. The Coris BioConcept is an example of the immune‐chromatography system used to detect OXA‐48 and KPC carbapenemases with almost 100% specificity and sensitivity. This system is relatively cheap, does not require technical know‐how, produces results within 2 min, and is readily available in the market (March‐rosselló, 2017).

##### Colorimetric methods

In this method, the test bacterium is incubated in the presence of an antibiotic. If the bacterium harbors the hydrolytic enzyme of the antibiotic, it hydrolyzes it and the pH of the medium changes, which is detected by a color change of an indicator. Usually, this test does not characterize the hydrolyzing enzymes but can detect variants of the enzymes that are expressed in a phenotypic analysis. Some kits used for detecting carbapenemases that are available in the market include RAPIDEC^®^ CARBA NP kit (bioMérieux) and the Rapid CARB Screen^®^ kit (Rosco Diagnostica A/S). They have a sensitivity and specificity of approximately 100% (Dortet et al., 2015).

##### MALDI‐TOF mass spectrometry

The MALDI‐TOF MS identifies bacteria, yeast, and filamentous molds in a couple of minutes using protein analysis (Rosselló & Pérez, 2016). During antibiogram, MALDI‐TOF MS predicts whether a bacteria harbors enzyme that hydrolyzes antibiotics, such as ESBLs and carbapenemases in approximately three hours. During this experiment, the test bacterium is incubated with the test antibiotic for some time, after which the medium is centrifuged and the supernatant is analyzed using the MALDI‐TOF MS. If the test is positive, the peak conforming to the antibiotic will disappear and new peaks conforming to the metabolites that result from the separation of the antibiotic will appear. If the test is negative, only the peak conforming to the antibiotic will appear. Chloramphenicol and clindamycin resistance can be predicted using this technique by detecting the 16S ribosomal RNA methylation executed by methyltransferases (Savic, Lovric, Tomic, Vasiljevic, & Conn, 2009).

### 4.4 The effects of the agricultural spread of resistance on human health and the economy

The dissemination and persistence of antibiotic resistance in the agricultural milieu signify a universal health crisis. It is estimated that, by 2050, antibiotic resistance will cause an annual increase in the death rate from 700,000 to 10 million and a cost increase of US $100 trillion (Jasovský, Littmann, Zorzet, & Cars, 2016). It is, therefore, imperative to have a full understanding of how antibiotic resistance thrives within the agricultural ecosystem and how they are spread to humans. Humans get infected by antibiotic‐resistant pathogens originating from the agricultural environment through direct contact with livestock or plant materials that are reservoirs of ARB (Chang, Wang, Regev‐yochay, Lipsitch, & Hanage, 2014). This might occur through the ingestion of farm produce, animal meats, or water contaminated with resistant pathogens.

In the same vein, genes that code for antibiotic resistance can also be dispersed to human pathogens through HGT via the food web. Once the human body is colonized by these pathogens and antibiotics are administered, antibiotic‐resistant lineages—which are naturally selected by these antibiotics—will begin to emerge and proliferate (Chang et al., 2014). Infections caused by antimicrobial‐resistant pathogens cause economic and clinical backlog to healthcare systems, government, patients, and their families (Golkar et al., 2014). About two million Americans develop health care‐associated infections due to antibacterial resistant pathogens, which eventually results in 99,000 deaths per year (Golkar et al., 2014). Significant expenses caused by antibiotic‐resistant infections usually overburden the healthcare system of a nation, and as such, caregivers are left with the option of dispensing antibiotics that are very toxic and expensive to their patients when the first‐line and second‐line antibiotics become scarce (CDC, 2013; Lushniak, 2014). Moreover, when patients come down with diseases instigated by antibiotic‐resistant pathogens, they are required to stay longer in the hospital, see the doctor more regularly, undergo extended healing periods, and might even have higher chances of long‐term debilities (CDC, 2013).

### 4.5 How to tackle environmental occurrence and spread of antibiotic resistance among clinically relevant pathogens

Globally, it is established that antibiotic resistance is a threat to the existence of humans and even animals; therefore, necessary measures need to be put in place to combat the menace caused by ARB and ARGs, starting at the environmental level. Because antibiotics at subtherapeutic levels modulate the evolution of antibiotic resistance among pathogens and commensals (Grenni et al., 2018), the prudent use of antibiotics in veterinary and agricultural sectors should be highly encouraged, as antibiotics should be limited to the treatment of animals using the right dose and duration and not for the promotion of growth. Based on these reasons, the EU implemented some level of restrictions on the use of antibiotics as growth promoters since 2006 (Kemper, 2008). Antibiotic regulations have also become more stringent in the United States (Chang et al., 2014). In 2012, the “German Federal Ministry of Agriculture, Food and Consumer Protection” announced the plans made to minimize the utilization of antimicrobials in animal farming (Rehder, 2012). In human medicine, only antibiotics prescribed by the physician should be used and the course must be completed even when symptoms subside. The reduction in the use of antibiotics can be encouraged when there is an improvement in the nutritional status, hygiene, and health status of humans and animals, development and availability of vaccines, fewer use antibiotics as growth promoters, and implementation of strict and targeted licensing (Tasho & Cho, 2016).

Another strategy is adequate treatment and disposal of animal effluent. Manure and WWTPs effluents' treatment should be improved using more advanced techniques. Better waste management augmented by government regulations should be implemented on most farms and municipal WWTPs. It is no longer news that the concentration of antibiotics present in manure can be drastically reduced using methods like anaerobic fermentation and composting.

Initiation of public media‐based awareness programs—such as documentaries, lectures, campaigns, online discussion forums, conferences, advertisements, short movies, poster presentation, animations, and lectures—will go a long way in updating people including children, farmers, physicians, veterinarians, and researchers on antibiotic resistance stewardship approaches (Tasho & Cho, 2016). In November 2015, WHO organized the first “World Antibiotic Awareness Week” to help the public learn more about antibiotics and encourage the governments to act. Such events will reduce the misuse of antibiotics generally, hence reducing their spread in the environment.

The magnitude of propagation of AR determinants among clinically significant bacteria caused by the existence of ARB and ARGs in the environment is still not clear. Nonetheless, the rate at which environments contaminated with ARB and ARGs affects the dissemination of AR can be determined with adequate biological risk assessment. Hence, control measures that involve management and policies can easily be adapted to curb antibiotic resistance proliferation (Berendonk et al., 2015). Combined risk assessment of the environmental development and occurrence of antibiotic resistance addresses the following concerns: first, the ability of antibiotics in their sublethal concentration to cause the evolution of ARB in intricate communities of bacteria; second, the extent of dissemination of ARGs from sections of the environment subjected to anthropogenic pressure such as wastewater treatment effluents, freshwater sources, manure and soil to human‐related pathogens and commensals; and finally, the transfer of these resistant pathogens to humans (Berendonk et al., 2015). For all of these to be achieved, it is imperative to describe antibiotic resistance in environment resistant strains, standardize resistance testing in samples collected from different sections of the environment, and establish a detailed database for all the data obtained from both environmental and clinical setting to ease the evaluation of the link that exist between antibiotic resistance in the environment and clinically significant bacteria (Berendonk et al., 2015).

## 5 CONCLUSION

We have presented an overview of the general knowledge of antibiotics, antibiotic resistance, and the possible transmission routes of antibiotics, ARB, and ARGs within different sections of the agro‐ecosystem. It will provide a knowledge base of antibiotic resistance “hotspots” in the agricultural environment and help to map out the prevalence of antibiotic resistance within the agricultural environment. It will also inform policymakers and aid antibiotic resistancesurveillance within the agro‐ecosystem. Improvement of sanitation and hygiene, restriction of nonmedical use of antibiotics, and adequate biological risk assessment remain the most critical strategies to curb the occurrence of antibiotic resistance within the agro‐ecosystem. A better understanding of hotspots and transmission routes is necessary for the source tracking of environmental contaminants and create a link between the source of contamination and their destination, which in most cases will be the food web (including fresh farm produce or animal meat). However, more studies are needed to estimate the amount of antibiotics that are discharged into the agricultural ecosystem and the quantitative estimate of human exposure to antibiotics, ARB, and ARGs. Additionally, further studies are required to address the knowledge gaps and access the potential risks—antibiotics, ARB, and ARGs in the environment pose to human well‐being and the broader environmental ecosystem. Moreover, the need for collaborations between veterinarians, physicians, farmers, researchers, and nongovernmental organizations to curb the menace of AR cannot be overemphasized.

## CONFLICT OF INTEREST

None declared.

## AUTHOR CONTRIBUTION

Chidozie Declan Iwu: Conceptualization (equal); Data curation (equal); Formal analysis (equal); Investigation (equal); Writing‐original draft (equal); Writing‐review & editing (equal). Lise Korsten: Conceptualization (equal); Funding acquisition (equal); Writing‐review & editing (equal). Anthony I Okoh: Conceptualization (equal); Methodology (equal); Supervision (equal); Writing‐review & editing (equal).

## 6 ETHICS STATEMENT

None required.

## Data Availability

Not applicable.
